# When We Meet in a Clearing: Making Research Accessible to Patients and Patient Experience Accessible to Clinicians

**DOI:** 10.1177/2374373518819441

**Published:** 2018-12-09

**Authors:** Giovanni Biglino, Sofie Layton, Jo Wray

**Affiliations:** 1 Bristol Heart Institute, Bristol Medical School, Bristol, United Kingdom; 2 Great Ormond Street Hospital for Children, NHS Foundation Trust, London, United Kingdom

**Keywords:** cardiovascular disease, pediatrics, patient perspectives/narratives, patient engagement

## The Patient: A Complex Case of Congenital Heart Disease

It was summer and Arif was undertaking a work experience with our cardiac engineering team. Arif was an 18-year-old patient with complex congenital heart disease and he had expressed an interest to see that other, hidden side of the hospital that he knew so well. By then he had already had a model of his heart made for a clinic appointment, and he had also taken part, with 6 other patients of similar age, in an artist-led creative workshop that we had devised and in which he had started to explore his narrative. He was a young man of few words and it was not explicit whether it was the mix of technical and creative work that sparked his curiosity. But he did e-mail us and we agreed on a 2-week visit.

During those days, we showed Arif how medical images can be processed and modeled, how magnetic resonance imaging (MRI) data are acquired, how different software programs work. And one day—in part also to offer him a more varied experience—we planned a follow-up to the creative workshop in which he had taken part, suggesting working 1:1 with the bioengineer (G.B.) and the artist (S.L.) to develop some of the ideas that had emerged in the previous workshop. He had previously talked of his purple body outline (referring to his cyanosis); when representing his inner landscape in the form of a body map, he had discussed why he would place his home in one foot and the hospital in the other, as grounding forces; he had gently discussed limitations to his lifestyle, such as food that cannot be eaten, but with impressive clarity and acceptance—allaying some concerns of the health-care team looking after him about whether he had grasped the severity of his condition. That day we thought that the focus could delicately shift from the whole unique individual to look more directly at the heart, as the topic that we wanted to explore, and that we had built sufficient trust and perhaps we could begin to look inside ourselves.

## The Workshop: Engagement and Re-Presentation

A room in the hospital was set up with creative outputs and notes from the previous workshop, heart models, heart outlines (including a 3-D rendering of Arif’s latest MRI). The tone was light, especially in the beginning, but in retrospect the three of us were all aware that we were about to embark on something special, with the artist leading both Arif and the bioengineer to conceive the image, the design, the metaphor that would represent their hearts.

If you were a toy from your childhood, what toy would you be? If you were an item of clothing, what would you be? If you were a flower, what flower would you be? Short sentences yet so revealing—wooden blocks, sunflowers, spinach leaves, lionesses, warm jumpers, wooly hats, all were clearly encapsulating so much more than the immediate imagery suggested. And then looking at hearts, their form, their lines, their sculptural quality, their anatomical landmarks. Whether 2-dimensional images or 3-dimensional models, every heart is similar to another yet also profoundly different, and some are entirely unique—or are they all unique? How do they work? How do they manage to keep the time of our lives? Why is that soft sound a bit louder and a moment later barely audible? Toward the end, we tried to recapitulate all those images that had come up in conversation, and the artist asked directly: if I were to make your hearts, what would they look like?

So there they were. Arif said: “My heart is like a patchwork, a puzzle, but some bits are missing; like a Rubik’s cube, where some of the facets are missing, a Rubik’s cube that cannot be resolved.” In effect a Rubik’s cube that is constantly moving—*click, click, click*—in search for a better configuration, in search of repair. And on the other side of the table, a wooden sculpture—the bioengineer knows it just as clearly—by Barbara Hepworth, fluid lines and inner surfaces painted in a light shade of green, looking in and looking out. Both so specific. We could almost see them in front of us.

We went away with a strong sense that something important had happened, something important had been shared. Arif finished his work experience. But those images and stories continued to reverberate. Upon reflection, it became clear: that would be our next project. That was the starting point. That was the essence of the research. We got a glimpse of something extraordinary and that merging of art and science became the heart of the matter.

We embarked on the journey. The research was funded. The project developed, exploring that dialogue between our medical heart and our metaphoric heart (or are they one and the same?). Tens of patients with different heart conditions (adolescents and adults with congenital heart disease, patients with arrhythmias, cardiomyopathy patients, parents of babies born with congenital heart disease) took part in a series of workshops and the conversation grew exponentially. And the metaphors and stories that were being shared also increased: symbols of resilience—fighting hearts, a female soldier, a woman warrior; natural forms—growing into blossoming trees, sweet and delicate like a strawberry; evocative images—echoing deep-sea sounds, whales singing, murmuring. In the background, the Rubik’s cube was constantly clicking, its clicking reminiscent of Arif’s mechanical heart valve.

Two years pass, the project has grown and is culminating in an exhibition touring the United Kingdom cities of Newcastle, Bristol, and London (www.insidetheheart.org). A time of anticipation and excitement, still a lot to do but also recognition of how far we have come. In the lead up to this, one evening, we get the news that Arif had died.

## The Clearing: Receiving and Sharing Stories

The image of the Rubik’s cube has become even more poignant, just as Arif’s role in the project becomes even clearer. It is not the theoretical idea of involving patients in research, with its associated boxes to fill in on grant application forms, although it absolutely reflects on the advocated importance of shaping research questions with patients. It is something more essential. It is, firstly, the importance of creating safe creative spaces and listening. It is also—it is clear now—what narrative scholars indicate as “honoring the stories of illness” (1). On the one hand, it is the realization that the work would have been different if Arif had not shared his story; perhaps similar, perhaps we would have had intuitions that would have brought us to a similar place, we cannot know, but certainly the journey would have been different. On the other hand, there is a sense of gratitude that becomes humbling.

What the work stemming from these conversations can do is hopefully engender a reflection, as part of a broader endeavor from other clinicians, other artists, other scientists, other patients. But together with the sense of celebration, there is also an equally strong awareness of what is left unsaid and unknown. In her essay *On being ill*, Virginia Woolf wrote: “We do not know our own souls, let alone the souls of others. Human beings do not go hand in hand the whole stretch of the way. There is a virgin forest in each; a snowfield where even the print of birds’ feet is unknown. Here we go alone, and like it better so. Always to have sympathy, always to be accompanied, always to be understood would be intolerable” (pp.11-12) (2). While we may not fully explore those lands, we can become even just a little bit more aware of those virgin forests. Interestingly, another metaphor used in the world of narrative medicine is that of the clearing (3), where now the forest is not our private forest, but the forest of health care, and where the clearing is that space where we can gather. So maybe that is what happened, back on that summer afternoon: we converged and met in a clearing, the three of us with our own stories and ready, in that moment, to share fragments of them.

**Figure 1.**
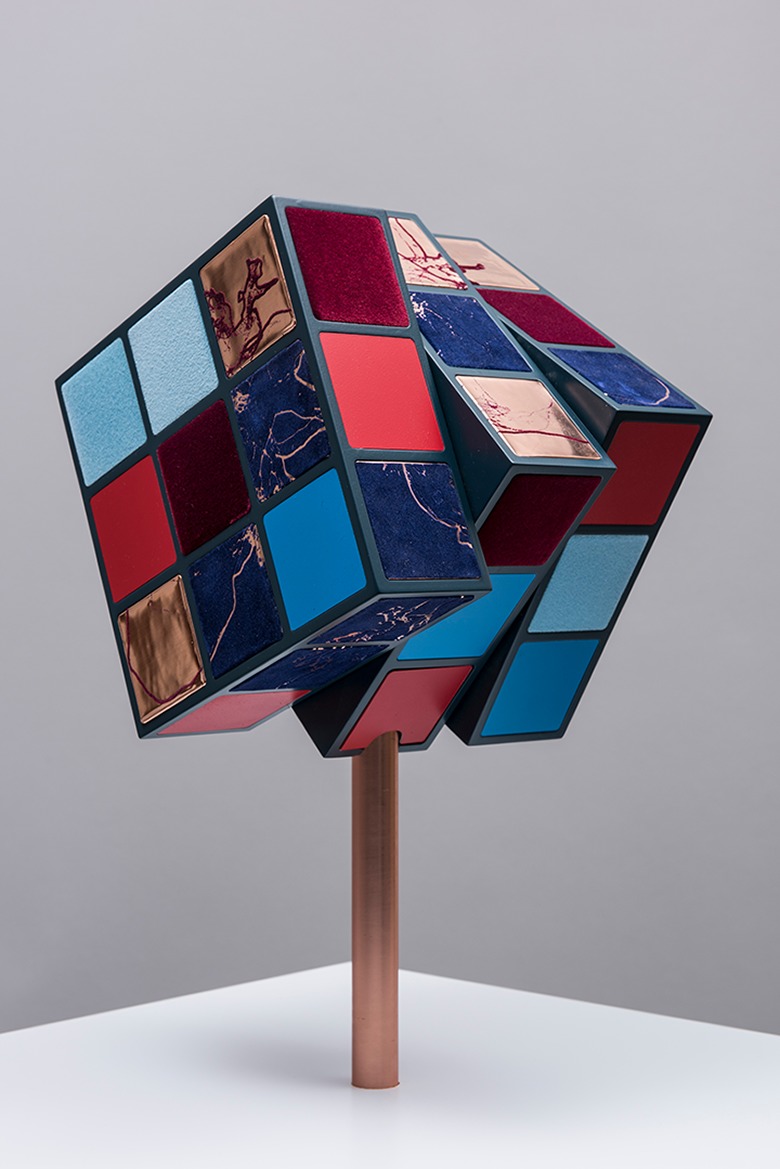
Sofie Layton, *Rubik’s Heart II*, 2018. The sculpture comprises a kinetic motor powering a cube made of copper, flocked and printed squares (21 cm edge length). The sculpture is presented as part of the heart of the matter exhibition (www.insidetheheart.org). Image by Stephen King.

Technologies such as 3-D printing and medical imaging came together with different arts practices, ultimately creating a body of original artworks for *The Heart of the Matter* exhibition, touring the United Kingdom in 2018. The opportunity is not only that of presenting the artwork, the complexity of cardiovascular anatomy, the sensitivities around heart transplant, the delicate encounters in hospital waiting rooms, to a general audience, but also to re-present all of this to clinicians, to enable them to get a glimpse of the world of the patient and understand what it means to be ill from their perspective, using words or images that do not easily arise in the clinical encounter. Through the artist’s filter, stories take new forms, and their essence is not only preserved but also distilled, reflecting on the fragility of life, the complexity of surgical procedures, or the implications of chronic conditions beyond the hospital space, allowing for different conversations to take place and for new learning to happen. As part of the exhibition, a kinetic sculpture of a Rubik’s Cube (Figure 1) clicks and turns but its facets keep failing to come together, still it cannot be resolved. While giving form to and honoring a very specific story, the sculpture abstracts the complexity of the surgical repair of congenital heart disease beyond the purely medical dimension.
